# Causal models and prediction in cell line perturbation experiments

**DOI:** 10.1186/s12859-024-06027-7

**Published:** 2025-01-07

**Authors:** James P. Long, Yumeng Yang, Shohei Shimizu, Thong Pham, Kim-Anh Do

**Affiliations:** 1 Department of Biostatistics, The University of Texas MD Anderson Cancer Center, Houston, TX USA; 2 Biomedical Informatics, The University of Texas Health Science Center at Houston, Houston, TX USA; 3 Faculty of Data Science, Shiga University, Hikone, Shiga Japan

**Keywords:** Causal inference, Prediction, Perturbation biology, Systems biology

## Abstract

In cell line perturbation experiments, a collection of cells is perturbed with external agents and responses such as protein expression measured. Due to cost constraints, only a small fraction of all possible perturbations can be tested *in vitro*. This has led to the development of computational models that can predict cellular responses to perturbations *in silico*. A central challenge for these models is to predict the effect of new, previously untested perturbations that were not used in the training data. Here we propose causal structural equations for modeling how perturbations effect cells. From this model, we derive two estimators for predicting responses: a Linear Regression (LR) estimator and a causal structure learning estimator that we term Causal Structure Regression (CSR). The CSR estimator requires more assumptions than LR, but can predict the effects of drugs that were not applied in the training data. Next we present Cellbox, a recently proposed system of ordinary differential equations (ODEs) based model that obtained the best prediction performance on a Melanoma cell line perturbation data set (Yuan et al. in Cell Syst 12:128–140, 2021). We derive analytic results that show a close connection between CSR and Cellbox, providing a new causal interpretation for the Cellbox model. We compare LR and CSR/Cellbox in simulations, highlighting the strengths and weaknesses of the two approaches. Finally we compare the performance of LR and CSR/Cellbox on the benchmark Melanoma data set. We find that the LR model has comparable or slightly better performance than Cellbox.

## Introduction

In cell line perturbation experiments, a collection of cells is perturbed with gene knockdowns, overexpression, or pharmaceutical drugs and responses such as cell survival and gene and protein expression are measured. The results of these experiments play an important role in our understanding of cellular biology and in development of treatments for complex diseases such as cancer [2–5].

There are a huge number of possible perturbations that can be applied to a cell line. For example, in human cell lines there are $\sim 20,000$ genes that could be perturbed (e.g. knocked out). Thus there are $\sim 200$ million perturbations of gene pairs (double knockouts). Further each perturbation may be applied across hundreds of cell lines (e.g. cells of different types of cancer). Thus in practice even large-scale experiments can only test a small fraction of all possible perturbations.

This limitation has led to the development of *in silico* perturbation response prediction models [1, 4, 6–10]. Models are typically trained on a set of perturbations that are experimentally tested in a laboratory and where cellular responses to the perturbation are known (up to technical replicate variability). These *in silico* models can then be used to make response predictions for untested perturbations. Predicted responses of biological interest, e.g. a perturbation which is predicted to suppress growth in a tumor cell line, can then be experimentally validated *in vitro*.

[1] proposed Cellbox, a perturbation prediction model based on a system of Ordinary Differential Equations (ODEs). Cellbox was benchmarked against several competitors on a Melanoma cell line in which cells were perturbed with 12 drugs, given at varying concentrations in each experiment. Cellbox achieved the best performance both on predicting cellular responses to drugs used in the training set and on predicting responses to drugs not used in the training set. This latter form of prediction, termed Leave One Drug Out (LODO) validation, is both challenging and scientifically impactful because it implies that the model can extrapolate to predict the effect of new drugs. In this work, we make the following contributions:

1. We propose a causal Structural Equation Model (SEM) for modeling the effect of drug perturbation on cell lines. From this model, we derive two estimators, Linear Regression (LR) and Causal Structural Regression (CSR), for predicting cellular responses to perturbations. These results show that CSR, which explicitly estimates the coefficients in a causal graph, can extrapolate to predict cellular responses to untested (in training set) drugs.
2. We derive analytic results which show that the linear version of Cellbox is equivalent to CSR. This provides a formal causal interpretation for the Cellbox model which was not discussed in [1].
3. We compare LR and CSR/Cellbox in simulations. These simulations demonstrated the strengths/weaknesses of the two approaches, including sensitivity of CSR/Cellbox to misspecification of the direct effect of interventions.
4. We show that LR has comparable or superior performance to Cellbox on the Melanoma benchmark data set. Our results reinforce the finding in other works that simple benchmark models may obtain equal or better performance than complex models in cell line perturbation response prediction [11].

All code and data for reproducing the results in this work is publicly available.^1^

## Overview of data, prediction problem, and connections to existing work

**Fig. 1:**
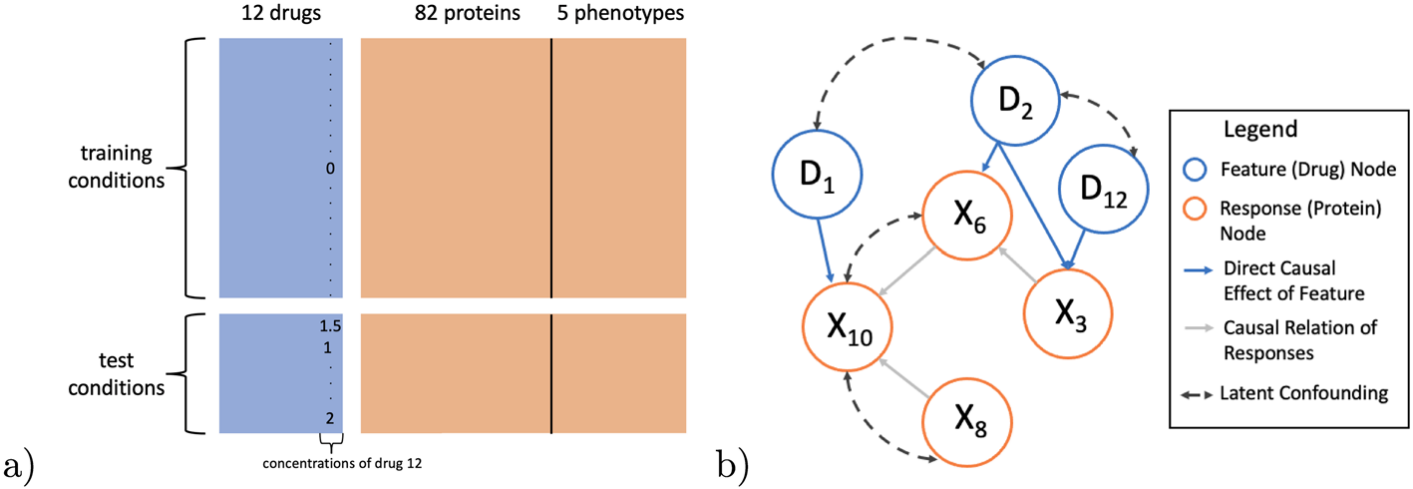
**a** Overview of perturbation data with a leave-one-drug-out (LODO) training / test set split. Drug 12 is never used in training. **b** Causal graphical model for subset of drugs and responses (proteins and phenotypes). Drugs are exogenous variables with known targets, e.g. it is assumed known that drug 12 directly effects protein $X_3$. Black dashed lines represent hidden confounding. For example unmeasured cell cycle may effect responses $X_{10}$ and $X_8$

In this work we consider perturbation experiments on a RAFi-resistant melanoma cell line SkMel-133 originally collected in [4]. The data structure is depicted in Fig. 1a. The cell line was treated with 89 drug perturbations (rows). Perturbations are defined by the concentrations of 12 drugs (12 columns of blue matrix). Drugs were applied as a single agent and in combinations of two drugs. Since at most two drugs were used in any experiment, each row of the blue matrix contains either 1 (if perturbation uses single drug) or 2 (if perturbation uses two drugs) non-zero values. In each perturbation experiment, the expression of 82 proteins was measured 24 h after perturbation using Reverse Phase Protein Arrays [12]. In addition, five cell phenotypes were measured, quantifying cell-cycle progression and cell viability (orange columns). For this work, we use the data as supplied by [1].^2^

In perturbation prediction, drug concentrations (blue columns) are used to predict protein and phenotype responses (orange columns). Data is divided into training and test sets. Using only the training data, a model is constructed which can predict protein/phenotypes from the drug concentrations. The performance of the model is then evaluated using the test set drug concentrations and protein/phenotypes. When the test set is a simple random sample of all perturbations, this setup matches the standard approach to fitting and validating predictive models. Models such as linear regression of response variables on the drug concentrations can be used.

In practice one would like to construct a model which can accurately predict the effect of untested drugs and in doing so identify perturbations with interesting responses for further follow up. Random Fold (RF) cross validation, in which the test conditions are a simple random sample of all conditions, does not represent this use case well because all drugs are used in training. A more challenging form of validation, leave-one-drug-out (LODO), more closely aligns with the intended scientific uses of the perturbation prediction model. Figure 1a depicts a LODO training-test set split. Here drug 12 is left out of the training set i.e. the concentration of drug 12 in the training data is always 0 because drug 12 was never used in training perturbations. The test perturbations all use drug 12 so column 12 of the drug matrix is never 0 in test. LODO prediction is challenging for regression models because there is no way for the model to determine the effect of drug 12 on the response variables. For example, coefficients in a linear regression of response on drugs will not be defined because the gram matrix is not invertible.

The direct targets of drugs are often known a priori. For example, a mitogen-activated protein kinase (MEK) inhibitor drug should directly reduce the expression of the MEK protein. Other changes in the system, could then be assumed to be a downstream effect of MEK inhibition. Using this information about drug targets, a causal model, which infers causal relations among the protein and phenotype response variables, can be used to predict responses in LODO validation. The approach is graphically summarized in Fig. 1b. For clarity only a small number of the drug and response variables are shown. Drugs (blue nodes) are known to target (blue arrows) particular proteins (orange nodes). For example drug $D_{12}$ targets protein $X_3$. The causal relations among proteins is unknown a priori (grey arrows). Training set perturbations can be used to identify and estimate causal effects among the proteins. Then the effect of an untested perturbation, e.g. drug 12, can be determined by first assuming that the direct effect of drug 12 will be on protein 3, and then propagating this effect through the inferred protein network.

### Related work and innovation

[1] developed an ordinary differential equation (ODE) model termed Cellbox and tested it on the Melanoma cell line data, both using RF and LODO validation. Cellbox outperformed all competing algorithms in both forms of model validation. In the following sections, we derive analytic results relating Cellbox to causal structure learning models, providing a new causal interpretation of Cellbox.

Several works have developed models for predicting responses to previously tested perturbations in new settings. For example, [7, 8], and [13] developed deep-learning autoencoder models to predict responses to previously untested perturbation-cell type combinations. [6] considered a similar problem and developed prediction model, SI-A, derived from the synthetic control literature in causal inference. [10] developed GEARS to predict the effect of double knockouts/knockdowns using *in vitro* responses to single knockouts/knockdowns.

In these works, test perturbations were applied to training conditions in different cell types or cell lines. These prediction problems are less challenging than the LODO Melanoma prediction because in LODO the held-out treatment has not been applied in the training set. These methods cannot be directly applied to the LODO setting. There is a large literature on inferring causal relations among genes, termed gene regulatory networks [14–18]. However these methods generally do not use interventional data to estimate the network. More importantly, these works view the regulatory network (graph) as the target for inference while in this work we are primarily interested in using the inferred network to make predictions about how interventions (drugs) will affect the system.

Environment-based causal estimators such as Invariant Causal Prediction (ICP) and the Causal Dantzig (CD) have been used to predict the effect of untested gene-knockdowns in yeast cell lines [19–21]. These methods assume the existence of different data collection environments, such as an observational environment where samples are collected without any perturbation and an interventional environment where samples are perturbed. Causal effects among the response variables are estimated based on the principle of invariance. These methods do not leverage information on the direct targets of interventions, which is used by CSR and Cellbox considered in this work.

## Causal perturbation model and estimators

Let $X \in \mathbb {R}^{p}$ be a vector of protein and phenotype responses (row of orange matrix in Fig. 1a) and $D \in \mathbb {R}^{q}$ be a vector of drug concentrations (row of blue matrix in Fig. 1a). There are *n* training observations $\{(D_i,X_i)\}_{i=1}^n$. The objective is to predict a test response $X^{te} \in \mathbb {R}^p$ when drugs $D^{te} \in \mathbb {R}^q$ are applied for an observation not in the training data. Subscripts *j* and *k* will be used to denote specific elements of *X* and *D*.

We propose a causal Structural Equation Model (SEM) for how drugs effect response variables and then derive two estimators using the model. Figure 1b contains a graphical representation of the model. Formally:

1 $$\begin{aligned} D&\leftarrow f_D \nonumber \\ \epsilon&\leftarrow f_\epsilon \nonumber \\ X&= AX + g(D) + \epsilon . \end{aligned}$$

The distribution $f_D$ models selection of drug concentrations to apply to cells. As indicated by black dashed lines in the figure, concentrations may be dependent. The term $\epsilon \in \mathbb {R}^{p}$ is a vector of errors with $\mathbb {E}[\epsilon ]=0$. Elements of $\epsilon$ may be correlated, representing hidden confounding among response variables *X*. Confounding may be caused by factors such as temperature, cell cycle, and variations in laboratory conditions. Figure 1b represents this hidden (latent) confounding with dashed lines connecting orange nodes. We assume $\epsilon \perp \!\!\!\!\perp D$ which is generally well justified because the choice of drug concentrations *D* is independent of the unmeasured factors modeled by $\epsilon$. $A \in \mathbb {R}^{p \times p}$ is a matrix of coefficients where $A_{jk}$ represents the causal effect of a one-unit change of $X_{k}$ on $X_{j}$. The term $g(D) \in \mathbb {R}^p$ represents the direct effects of drug concentrations *D* on the response variables (protein concentrations).

In this work, we assume that $(I-A) \succ 0$ (positive definite). By Sylvester’s criteria, $I-A \succ 0$ whenever *A* represents an directed acyclic graph (DAG). Since $I-A \succ 0$ implies $I-A$ is invertible, Equation (1) may be rewritten as

2 $$\begin{aligned} X = (I-A)^{-1}g(D) + \underbrace{ (I-A)^{-1} \epsilon }_{\equiv \delta }. \end{aligned}$$

The prediction target is the mean response when drug concentrations $D \in \mathbb {R}^q$ are applied to the system:

3 $$\begin{aligned} f(D) \equiv \mathbb {E}[X|do(D)] = \mathbb {E}[X|D] = (I-A)^{-1} g(D). \end{aligned}$$

The equality between $\mathbb {E}[X|do(D)]$ and $\mathbb {E}[X|D]$ is justified by the fact that there are no backdoor paths from *D* to *X* [22, 23]. Note that this is only true for the vector $D \in \mathbb {R}^q$. For any individual drug concentration, e.g. $D_{12}$, latent confounding between drugs may induce backdoor paths. We now discuss two approaches to estimating $f$ and their relative strengths and weaknesses.

### Regression

One can assume $f$ belongs to a class of functions $\mathcal {F}$ and then select an *f* which minimizes loss. For example consider

4 $$\begin{aligned} \widehat{f} = {\underset{f\in \mathcal {F}}{\text {argmin}} \, } \sum _{i=1}^n ||X_i - f(D_i)||_2^2 + \gamma (f,\lambda ) \end{aligned}$$

where $\gamma$ is a penalty function and $\lambda$ controls the degree of penalization with $\gamma (f,0)=0$. The predicted response for the test observation is $\widehat{f}(D^{te})$. In the case where the direct effect of drugs on phenotypes is linear, i.e. $g(D) = BD$ for some $B \in \mathbb {R}^{p \times q}$, then the class of functions $\mathcal {F}$ is linear because $f(D) = (I-A)^{-1}BD$ and can be parameterized by $R = (I-A)^{-1}B$. In this case, the objective function may be written as

5 $$\begin{aligned} \widehat{R} = {\underset{R}{\text {argmin}} \, } \sum _{i=1}^n ||X_i - RD_i||_2^2 + \gamma (R,\lambda ) \end{aligned}$$

with predicted response

$$\begin{aligned} \widehat{X}_{LR} = \widehat{R}D^{te}. \end{aligned}$$

We term $\widehat{X}_{LR}$ the Linear Regression (LR) prediction.

For LODO validation with drug *j* held out, recall that $D_{ij}=0$ for all $i \in \{1,\ldots ,n\}$ (the training data), but $D^{te}_j \ne 0$. In this case, the regression estimators will not be consistent. For LR (Equation (5)), without regularization ($\lambda =0$), the minimizer of the objective function is unique if and only if

$$\begin{aligned} \begin{pmatrix} D_1^T\\ \vdots \\ D_n^T \end{pmatrix} \end{aligned}$$

is full rank. This will not be the case with LODO validation because every element of the $j^{th}$ column is 0. If sparsity inducing regularization is used, e.g. $\gamma (R,\lambda ) = \lambda \sum |R_{jk}|$, then the $j^{th}$ column of $\widehat{R}$ will be 0. In this case, the effect of drug *j* on all response variables is estimated to be 0 and hence inconsistent. Qualitatively, regression is unsuitable because LODO requires an extreme form of extrapolation where feature *j* (drug concentration *j*) is always 0 in training but non-zero in the test set.

### Causal structure regression (CSR)

Suppose $g$ (the direct targets and strength of effects of the drugs) is known. In this case, it is possible to estimate *A* (or equivalently $(I-A)^{-1}$). One possible estimator of *A* is obtained by regressing *X* on $g(D)$. Specifically

6 $$\begin{aligned} \widehat{A} = {\underset{\{A : I-A \succ 0 \}}{\text {argmin}} \, } \sum _{i=1}^n ||X_i - (I-A)^{-1}g(D_i)||_2^2 + \gamma (A,\lambda ). \end{aligned}$$

Consistency of this estimator does not require non-gaussianity, equal variance, or no hidden confounding assumptions common in the causal discovery literature [24–26]. Instead, the interventions (drug concentrations *D*) act as exogeneous variables which identify the causal structure matrix *A*. The predicted response to drug concentrations $D^{te}$ is

$$\begin{aligned} \widehat{X}_{CSR} = (I-\widehat{A})^{-1}g(D^{te}). \end{aligned}$$

We term $\widehat{X}_{CSR}$ the Causal Structure Regression (CSR) prediction because the prediction is based on a regression estimate of the causal structure *A*. The *j*, *k* element of matrix $(I-A)^{-1}$ is the total effect of response $X_k$ on $X_j$. In practice, it may be simpler to directly estimate $T = (I-A)^{-1}$, check whether the resulting $\widehat{T}$ is positive definite, and then use the estimate $\widehat{T}$ to predict $\mathbb {E}[X|D]$. When implementing this approach without regularization we have

7 $$\begin{aligned} \widehat{T} = {\underset{T \in \mathbb {R}^{p \times p}}{\text {argmin}} \, } \sum _{i=1}^n ||X_i - Tg(D_i)||_2^2 \end{aligned}$$

and

8 $$\begin{aligned} \widetilde{X}_{CSR} = \widehat{T}g(D^{te}). \end{aligned}$$

Now consider CSR for estimating $f(D^{te})$ with LODO validation with drug *j* held out. The estimator $\widehat{T}$ (Equation (7)) will be uniquely defined if and only if

$$\begin{aligned} \begin{pmatrix} g(D_1)^T \\ \vdots \\ g(D_n)^T \\ \end{pmatrix} \end{aligned}$$

is full column rank. This rank condition may be satisfied even in the LODO setting. In particular, the rank condition for CSR requires that the training drugs directly target every response variable, not that every drug is used in the training set.

The relative merits of the Regression and CSR predictions are summarized as follows:

- **LODO Validation**: As discussed, regression is inconsistent for LODO validation.
- **p versus q**: CSR estimates *A*, which consists of $p^2$ parameters corresponding to direct effects of all response variables on each other. LR estimates *R*, which consists of *qp* parameters corresponding to the total effect of each of the *q* drugs on the *p* response variables. Thus when $q < p$, regression requires estimating fewer parameters.
- *g* **Assumption**: CSR requires knowledge of *g*, the direct effects of interventions on response variables. If *g* is unknown or contains a large amount of uncertainty, the regression estimator may be preferred.
- **Interpretability:** CSR is more interpretable because it estimates matrix *A* which encodes how response variables causally effect each other, providing biological insight on how cells function.

## Causal structure regression and cellbox

We now discuss Cellbox, an ODE model introduced in [1], which obtained state-of-the-art prediction performance on the Melanoma cell line perturbation experiments. First we summarize the Cellbox modeling and fitting procedure, modifying notation in certain instances for clarity.^3^ We then derive results which demonstrate a close connection between Cellbox and CSR.

Cellbox uses a system of ODEs to model how proteins and phenotypes influence each other across time. Let $x_i(t,\theta ) \in \mathbb {R}^p$ be the log-normalized change at time *t* (relative to time 0) of a set of *p* proteins and phenotypes under perturbation condition *i*. The unknown parameters $\theta$ control how proteins influence each other. For observation *i*, drug concentrations $D_i \in \mathbb {R}^q$ are applied. Define $u_i = g(D_i)$, the direct effect of applying drug concentrations $D_i$ to the system. Since $D_i$ and *g* are assumed known, $u_i$ is known as well.

Response *j* (protein or phenotype) under condition *i* is modeled by

9 $$\begin{aligned} \frac{\partial x_{ij}(t,\theta )}{\partial t} = \tau _j \phi \left( \sum _{k \ne j}w_{jk}x_{ik}(t,\theta ) + u_{ij}\right) + w_{jj} x_{ij}(t,\theta ). \end{aligned}$$

The unknown model parameter is $\theta = (W,\tau )$ with $w_{jk}$ for $j \ne k$ representing the interaction between $x_j$ and $x_k$, $w_{jj}$ characterizes the effect of decay (the tendency of protein *j* to return to the original level before perturbation), and $\tau _j$ controls the saturation effect of the protein. Cellbox can be fit with several envelope functions $\phi$ including identity, clipped linear, and sigmoid.

In [1] Cellbox was fit with response variables measured at a single time point 24 h after perturbation initiation. It was assumed that by this time, the system has reached steady state. The steady state (equilibrium) changes implied by the model is

10 $$\begin{aligned} x_{ij}(\theta ) \equiv \lim _{t \rightarrow \infty } x_{ij}(t,\theta ). \end{aligned}$$

To estimate parameters $\theta$, discrepancy between model predicted responses ($x_{ij}(\theta )$) and experimental responses ($X_{ij}$) was computed with a $L_1$ (lasso) penalty term to induce sparsity on the off-diagonal elements of *W*. Specifically

$$\begin{aligned} L(\theta ) = \sum _{j=1}^p \sum _{i=1}^n |X_{ij} - x_{ij}(\theta )|^2 + \lambda ||W - diag(W)||_1 \end{aligned}$$

where $diag(W) \in \mathbb {R}^{p \times p}$ is the diagonal component of *W*. Given a candidate $\theta$, an ODE solver can be used to approximate $x_{ij}(\theta )$. Subsequently, $\theta$ are updated using gradient descent with automatic differentiation to determine

11 $$\begin{aligned} \widehat{W},\widehat{\tau } = {\underset{\theta =(W,\tau )}{\text {argmin}} \, } L(\theta ). \end{aligned}$$

To predict the response $X^{te}$ for some combination of drugs $D^{te}$, first the direct effects of the drug are determined ($u_i = g(D^{te})$), followed by an ODE solver to approximate steady state expression levels (Equation (10)) using parameters $\widehat{W}$ and $\widehat{\tau }$.

We now show that using a linear envelope function $\phi$ and setting $\tau =1$, Cellbox is equivalent to the linear CSR model.

### Theorem 1

Suppose $\phi$ is a linear envelope function, $\tau =1$, and $W \prec 0$. We have the following results

1. The equilibrium state of the Cellbox model (Equation (10)) has closed form
  12 $$\begin{aligned} x_i(\theta ) = (x_{i1}(\theta ),\ldots ,x_{ip}(\theta ))^T = -W^{-1}g(D_i) \end{aligned}$$
  and the Cellbox predicted response for test drugs $D^{te}$ is
  13 $$\begin{aligned} \widehat{X}_C = -\widehat{W}^{-1}g(D^{te}). \end{aligned}$$
2. The Cellbox parameter optimization (Equation (11)) may be expressed as
  14 $$\begin{aligned} \widehat{W} = {\underset{W : W \prec 0}{\text {argmin}} \, } \sum _{i=1}^n ||X_i - (-W^{-1})g(D_i)||_2^2 + \lambda ||W - diag(W)||_1. \end{aligned}$$
3. If the penalty function $\gamma (A,\lambda ) = \lambda ||A-diag(A)||_1$ is used in CSR in Equation (6), then
  15 $$\begin{aligned} \widehat{A} = \widehat{W} + I \end{aligned}$$
  and $$\widehat{X}_{CSR} = \widehat{X}_C$$.

See Section A.1 for a proof. Equations (12) and (14) show that for the linear version of Cellbox ($\phi$ identity and $\tau =1$), ODE solvers are not necessary for estimating parameters $\widehat{W}$ and making predictions. Equation (15) shows that linear Cellbox is a reparameterized version of CSR and test predictions $\widehat{X}_{C}$ and $\widehat{X}_{CSR}$ are identical. This provides a causal interpretation for Cellbox in terms of structural equations. The general principal of this result, that causal structural equation models are steady state limits of dynamical systems, has been derived in several previous works [27, 28]. The assumption that $W \prec 0$ is weak because 0 or positive eigenvalues in *W* imply the system is not converging to any steady state.

We note that the implementation of Cellbox in [1] set elements of *W* to 0 which represent phenotype to protein causal effects. This is accomplished by restricting the domain of the parameter optimization in Equation (11). This enforces the domain knowledge that proteins may influence phenotypes but not vice versa. For clarity of exposition, we do not impose the conditions here or in the simulations since they are not directly relevant for understanding the relationship between regression and causal predictive models. However in the application to the Melanoma cell line, we follow [1] and impose the restriction.

## Simulation

**Fig. 2:**
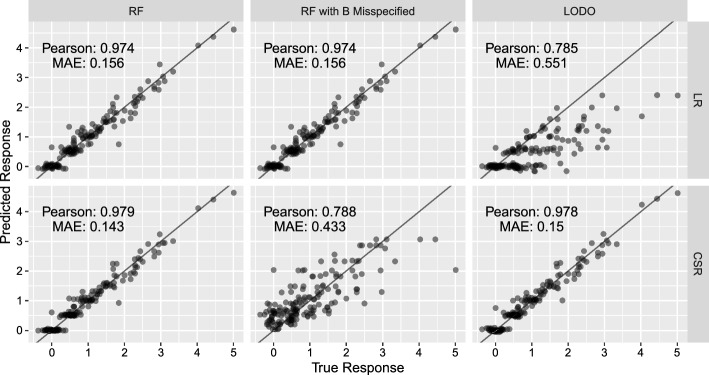
Comparison of performance of LR and CSR/Cellbox on simulated data. The x-axis is the true response and the y-axis is the predicted response. LR and CSR perform similarly for RF validation. For RF with *B* misspecified, CSR, which uses *B*, performs poorly. LR is unaffected by misspecified *B* because *B* is not used in the LR estimator. For LODO validation, LR performs poorly because it cannot model the effect of the left out drug on the responses

We conduct a simulation to compare the performance of the regression estimator ($\widehat{X}_{LR}$) and CSR/Cellbox ($\widetilde{X}_{CSR}$). We simulate from Causal SEM (1)

$$\begin{aligned} X = AX + g(D) + \epsilon \end{aligned}$$

using $p=5$ response variables ($X \in \mathbb {R}^5$) and $q=15$ drugs ($D \in \mathbb {R}^{15}$). Drugs are assumed to have a linear effect on response variables so $g(D) = BD$. The structure of *B* and *A* are specified in Equation (16). Five drugs target a single response variable and 10 drugs target two of the response variables. Drugs with a single target have a strength of 1 while drugs with 2 targets have a strength of 0.5 for each target. All possible combinations of 2 drugs are applied to the system, thus there are a total of $n= {15 \atopwithdelims ()2} = 105$ observations with each $D_i$ having exactly two non-zero entries equal to 1. The variable $X_1$ has a causal effect of 1.6 on $X_2$ and 1.2 on $X_3$. The variable $X_3$ has a causal effect of 2 on $X_4$. All other causal effects among the response variables are 0. The exogenous error $\epsilon$ is distributed $N(0,0.1^2)$.

16 $$\begin{aligned} B =\left( \begin{array}{llllllllllll} 1 & 0 & 0 & 0 & 0 & 0.5 & 0.5 & 0.5 & 0.5 & 0 & \cdots & 0 \\ 0 & 1 & 0 & 0 & 0 & 0.5 & 0 & 0 & 0 & 0.5 & \cdots & 0\\ 0 & 0 & 1 & 0 & 0 & 0 & 0.5 & 0 & 0 & 0.5 & \cdots & 0\\ 0 & 0 & 0 & 1 & 0 & 0 & 0 & 0.5 & 0 & 0 & \cdots & 0.5\\ 0 & 0 & 0 & 0 & 1 & 0 & 0 & 0 & 0.5 & 0 & \cdots & 0.5 \end{array}\right) \in \mathbb {R}^{5 \times 15} \, \, \, \, \, \, \, \, \, \, A = \left( \begin{array}{lllll} 0 & 0 & 0 & 0 & 0\\ 1.6 & 0 & 0 & 0 & 0\\ 1.2 & 0 & 0 & 0 & 0\\ 0 & 0 & 2 & 0 & 0\\ 0 & 0 & 0 & 0 & 0 \end{array}\right) \end{aligned}$$

Since $q > p$ (number of drugs is greater than number of response variables), regularization is not necessary ($\lambda$ is set to 0 in all the simulations). We fit the LR and CSR estimators under three settings:

- **Random Fold (RF):** The data is divided randomly into 2/3 training and 1/3 test. Since the training-test set split is random, every drug is used in training.
- **RF with B Misspecified:** The training-test set split is identical to RF. However the *B* matrix (direct effect of drugs) is misspecified. Instead of using the correct *B*, the 10 drugs with 2 targets are assumed to influence their targets with a strength of 1 (rather than the correct value of 0.5).
- **Leave-one-drug-out (LODO):** For each condition in the test set, one (of the two) drugs used in the condition is selected at random. The 2/3 of the training data is subset to only use conditions where the selected drug is not used. For LR, the coefficient on the held out drug is set to 0.

Results are summarized in Fig. 2. The true response values are plotted on the x-axis and the predicted response values are plotted on the y-axis. High correlations imply that the estimator is performing well in the respective setting. For Random Fold (RF) cross validation, both LR and CSR perform well. In the RF with B Misspecified setting, LR performs well and in fact makes identical predictions to RF validation because the LR estimator does not depend on *B*. In contrast, CSR performs poorly because it uses an incorrectly specified *B*. Finally in LODO, LR performs poorly because it incorrectly infers that the effect of the left out drug on all response variables 0. In contrast, CSR performs well because it models the causal relations among the response variables which enables it to generalize predictions to untested drugs.

**Fig. 3:**
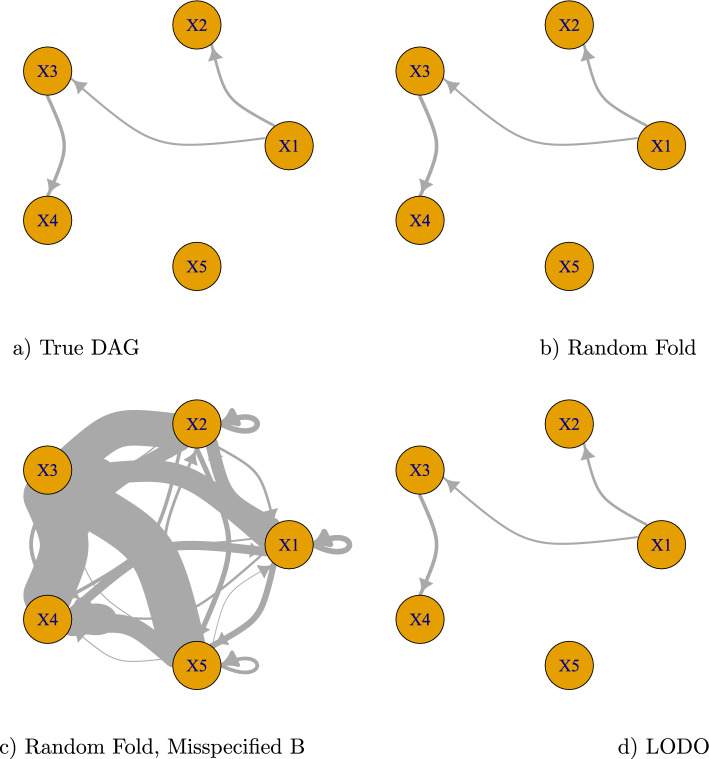
True network and estimated networks for different simulation settings. For Random Fold **b** and LODO **d**, the estimated *A* is quite close to the true DAG *A* **a**. For Random Fold with Misspecified *B* **c**, the estimated *A* contains many erroneous edges

The true *A* and the estimated $\widehat{A}$ for each setting (RF, RF with B Misspecified, LODO) are displayed graphically in Fig. 3. Note that CSR in LODO estimates *A* for each test observation. We plot only one of them here. Edge widths are proportional to size of the coefficient estimate. For visual clarity, small effects (coefficients less than 0.2 in absolute size) are not displayed. The Random Fold $\widehat{A}$ in Fig. 3b and the LODO $\widehat{A}$ in Fig. 3d closely resemble the true *A* in Fig. 3a. In contrast, Fig. 3c shows that when *B* is misspecified the resulting $\widehat{A}$ is a poor estimate.

## Melanoma cell line perturbation prediction

We compare Cellbox and LR for prediction of protein and phenotype responses in the Melanoma data set introduced in Sect. . The two validation procedures we describe below follow the procedures in [1]. Cellbox is implemented with a sigmoid activation function. See [1] for details on tuning parameter selection, choice of *g*, and the (causal) graph/network estimated by the model (Figure 5).

### Random fold cross validation

**Fig. 4:**
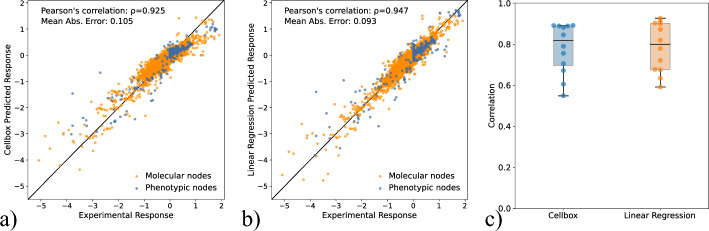
LR outperforms Cellbox in Random Fold (RF) CV while the two methods have similar performance in LODO. **a** Cellbox performance on Random Fold CV **b** LR performance on Random Fold CV **c** Comparison of performance of Cellbox and LR on LODO validation

The 89 experimental conditions are split into 70% training (62 conditions) and 30% testing (27 conditions). Models are fit on the training set and used to predict the responses on the test set. This process is repeated 1000 times and the predictions averaged across these runs. Predicted responses versus experiment responses are plotted for Cellbox in Fig. 4a and LR in Fig. 4b. LR predictions show a stronger correlation with the response than Cellbox (Pearson’s correlation of 0.947 versus 0.926) and lower mean absolute error (0.093 versus 0.105). RF cross validation favors regression models (relative to LODO) because the regression model estimates fewer parameters and does not require regularization.

### Leave one drug out

We now consider the more challenging Leave One Drug Out (LODO) validation where a drug is held out of training. For example, if the drug aMEK is held out, the training data is all conditions with aMEK concentration equal to 0 and the test set is all conditions where aMEK has been applied, either as monotherapy or in combination with other drugs. Since there are 12 drugs, there are 12 training-test set pairs.

For LR, we set the coefficient for the left out drug to 0 and fit the unregularized estimator to the remaining columns of $\textbf{D}$. This approach does not require tuning parameter selection. Qualitatively this assumes that the drug held out has no effect on any of the response variables. This represents a crude benchmark model rather than an empirically motivated model assumption.

For each test set, we compute the correlation between the true responses and the predicted responses. This results in 12 correlations for Cellbox and Linear Regression. Figure 4c displays these correlations. LR (mean correlation coefficient 0.784) and Cellbox (mean correlation coefficient 0.780) have similar performance.

## Discussion

The field of causal inference has historically focused on parameter estimation and hypothesis testing. Recently, several works have explored using causal models for prediction [1, 21, 29, 30]. Prediction performance is an important metric for measuring the quality of causal models in many scientific applications, including cell line perturbation experiments. For causal models to provide meaningful scientific insight, it is critical to understand their relationship with regression estimators and appropriately benchmark models when assessing performance.

Here we proposed a causal SEM for modeling responses in cell line perturbation experiments. We derived two estimators based on this model, LR and CSR. We derived analytic results demonstrating a close relationship between CSR and a recently proposed prediction model, Cellbox. The analytic results and simulations facilitated an improved understanding of the strengths and weaknesses of the two approaches to prediction. In brief, regression models, such as LR, are simpler to fit but lack an ability to extrapolate to new data settings, such as prediction of response to a drug not used in the training set. Causal modeling (CSR/Cellbox) is sensitive to the assumption of that the direct targets of the perturbations are known, making it most suitable for knockdown/knockout interventions with fewer off target effects than drug interventions.

Cellbox obtained state of the art performance on a Melanoma cell line perturbation data set, outperforming a Belief Propagation algorithm, a deep learning Neural Network (NN), and a co-expression model. Here we demonstrated that Cellbox, and hence all the competitor methods, failed to outperform LR in either RF or LODO validation. The latter finding is particularly surprising because this is a setting which favors causal modeling approaches. These results highlight that simple modeling strategies can be the most effective and are critically important when benchmarking performance of new models.

The Melanoma perturbation data set used here is relatively small, lacking any information on the temporal dynamics of responses to perturbations. Larger perturbation experiments test hundred or thousand of perturbations across dozens of cell lines with responses measured at multiple time points [2, 3, 31]. These data sets are likely to be more favorable to a model such as Cellbox, as they may contain sufficient information to identify and constrain model parameters. A recent generalization of Cellbox to simulated perturbations with responses measured across time showed promising performance [32].

## Data Availability

All data and code to reproduce results in this work are available at https://github.com/longjp/causal-pred-drug-code

## Appendix A Proofs

### A.1 Proof of Theorem 1

1. With the identity envelope $\phi (\cdot ) = \cdot$ and $\tau _i = 1$, Equation (9) simplifies to
  A1 $$\begin{aligned} \frac{\partial x_i(t,\theta )}{\partial t} = Wx_i(t,\theta ) + g(D_i). \end{aligned}$$
  Now rewrite the ODE to explicitly include drug effects in the system as time constant terms. Define
  $$\begin{aligned} y_i(t,\theta ) = \begin{pmatrix} g(D_i) \\ x_i(t,\theta ) \end{pmatrix} \in \mathbb {R}^{2p} \end{aligned}$$
  where
  A2 $$\begin{aligned} \frac{\partial y_i(t,\theta )}{\partial t} = \underbrace{\begin{pmatrix} 0 & 0\\ I & W \end{pmatrix}}_{\equiv A \in \mathbb {R}^{2p \times 2p}} y_i(t,\theta ) = \begin{pmatrix} 0\\ Wx_i(t,\theta ) + g(D_i) \end{pmatrix}. \end{aligned}$$
  There is a closed form solution for the system at time *t*, specifically
  $$\begin{aligned} y_i(t,\theta ) = e^{At}y_i(0,\theta ). \end{aligned}$$
  See [33] (Section 9.5 Theorem 2) for a derivation of this result. Further by Lemma 2, if *W* is invertible then,
  $$\begin{aligned} y_i(\theta ) \equiv \lim _{t\rightarrow \infty } y_i(t,\theta ) = \begin{pmatrix} g(D_i) \\ -W^{-1}g(D_i) \end{pmatrix}. \end{aligned}$$
  Thus $$x_i(\theta ) = -W^{-1}g(D_i)$$.
2. We have
  $$\begin{aligned} \widehat{W}&= {\underset{W}{\text {argmin}} \, } \sum _i \sum _j |X_{ij} - x_{ij}(\theta )|^2 + \lambda ||W - diag(W)||_1\\&= {\underset{W}{\text {argmin}} \, } \sum _i ||X_{ij} - x_i(\theta )||^2_2 + \lambda ||W - diag(W)||_1\\&= {\underset{W}{\text {argmin}} \, } \sum _i ||X_{ij} - (-W^{-1})g(D_i)||_2^2 + \lambda ||W - diag(W)||_1. \end{aligned}$$
3. Recall that the Cellbox and CSR optimization problems are
  $$\begin{aligned} \widehat{W}&= \underbrace{{\underset{\{W : W \prec 0\}}{\text {argmin}} \, }}_{\equiv S_1} \sum _{i=1}^n \underbrace{||X_i - (-W^{-1})g(D_i)||_2^2 + \lambda ||W - diag(W)||_1}_{\equiv h_1(W)}\\ \widehat{A}&= \underbrace{{\underset{\{A : I-A \succ 0 \}}{\text {argmin}} \, }}_{\equiv S_2} \underbrace{\sum _{i=1}^n ||X_i - (I-A)^{-1}g(D_i)||_2^2 + \gamma (A,\lambda )}_{\equiv h_2(A)} \end{aligned}$$
  Note that $h_1(W) = h_2(I + W)$ and $W \in S_1 \Leftrightarrow I + W \in S_2$. Therefore $\widehat{A} = \widehat{W} + I$. Finally
  $$\begin{aligned} \widehat{X}_C = - \widehat{W}^{-1}g(D^{te}) = (I - \widehat{A})^{-1}g(D^{te}) = \widehat{X}_{CSR}. \end{aligned}$$

### A.2 Proof of Lemmas

#### Lemma 2

$$\begin{aligned} \lim _{t \rightarrow \infty } e^{At} = \begin{pmatrix} I & 0\\ -W^{-1} & 0 \end{pmatrix}. \end{aligned}$$

#### Proof

Note that

$$\begin{aligned} e^{At}&= I + \sum _{i=1}^\infty \frac{A^it^i}{i!}\\&= I + \sum _{i=1}^\infty \frac{\begin{pmatrix} 0 & 0\\ W^{i-1} & W^i \end{pmatrix}t^i}{i!}\\&= \begin{pmatrix} I & 0\\ \sum _{i=1}^{\infty } \frac{W^{i-1}t^i}{i!} & \sum _{i=0}^\infty \frac{W^it^i}{i!} \end{pmatrix}\\&= \begin{pmatrix} I & 0\\ W^{-1}(e^{Wt}-I) & e^{Wt} \end{pmatrix}. \end{aligned}$$

We now show that $\lim _{t \rightarrow \infty } e^{Wt} = 0$ which implies the desired result. Let $c_j$ for $j=1,\ldots ,p$ be an eigenbasis for *W* such that $W c_j = \lambda _j c_j$. Note that since $W \prec 0$, $\lambda _j < 0$ for all *j*. Consider any $r \in \mathbb {R}^p$ with basis decomposition $r = \sum _j \gamma _j c_j$. We have

$$\begin{aligned} e^{Wt}r&= \sum _{j=1}^p \left( \sum _{i=0}^\infty \frac{W^it^i}{i!}\right) \gamma _j c_j\\&= \sum _{j=1}^p \left( \sum _{i=0}^\infty \frac{\lambda _j^it^i}{i!}\right) \gamma _j c_j\\&= \sum _{j=1}^p e^{\lambda _j t}\gamma _j c_j\\&\rightarrow 0. \end{aligned}$$

Since this is true for any *r*, $\lim _{t \rightarrow \infty } e^{Wt} \rightarrow 0$. $\square$

## Footnotes

1 https://github.com/longjp/causal-pred-drug-code

2 Available at https://github.com/sanderlab/CellBox.

3 See Model Configuration section of METHOD DETAILS in Star Methods of [1] for original exposition of model.
